# Pregnancy complications in G20210A mutation carriers associated with high prothrombin activity

**DOI:** 10.1186/s12959-021-00289-4

**Published:** 2021-06-05

**Authors:** M. G. Nikolaeva, A. P. Momot, M. S. Zainulina, N. N. Yasafova, I. A. Taranenko

**Affiliations:** 1 Altai Branch of FSBI “National Research Center for Hematology”, Barnaul, Russia; 2 FSBEI of Higher Education “Altai State Medical University”, 40 Lenina Ave, Barnaul, 656038 Russia; 3 Saint Petersburg State-Financed Health Institution “Birth Centre № 6 named after Professor V.F. Snegireva”, St Petersburg, Russia; 4 Obstetrics, Gynecology and Reproductive Medicine Department “Pavlov First Saint Petersburg State Medical University”, St Petersburg, Russia; 5 Altai Regional Clinical Hospital, Barnaul, Russia

**Keywords:** Factor II activity, Prothrombin G20210A mutation, Preeclampsia, Fetal growth retardation, ROC analysis

## Abstract

**Objective:**

To study the association between high activity of Factor II (prothrombin) in blood plasma with G20210A mutation and the development of great obstetrical syndromes.

**Material and methods:**

A prospective clinical cohort study was conducted on 290 pregnant women (average age 31.7 ± 4.7 years old). The main group was made up of 140 G20210A patients, while the control group comprised 150 women with the *wild G20210G* type. The aim was to evaluate the activity of Factor II in the venous blood plasma during the stages of pregnancy with regard to trophoblast invasion waves. As per results, association analysis of Factor II activity value and gestational complications was carried out.

**Results:**

In the control group, the median (Me) of Factor II activity ranged from 108% (preconception period) to 144% (pregnancy) [95% CI 130–150]. In patients with the GA type, the value was significantly higher in related periods, ranging from 149 to 181% [95% CI 142–195], *p* < 0.0001. With Factor II activity ranging from 148.5 to 180.6%, pregnancies in the main group had no complications. Higher levels of Factor II activity were associated with the development of early and/or severe preeclampsia (PE) and fetal growth retardation (FGR).

**Conclusion:**

The data obtained regarding Factor II activity in blood plasma, juxtaposed with the development of great obstetrical syndromes, allow to assume that manifestation of G20210A in early and/or severe PE and FGR is associated with this coagulation factor’s level of activity. Threshold value of the Factor II activity with G20210A mutation, allowing to predict the development of PE, comprised 171.0% at the preconception stage (AUC – 0.86; *p* < 0.0001) and within 7–8 weeks of gestation it was 181.3% (AUC – 0.84; p < 0.0001).

## Introduction

Prothrombin G20210A mutation was first described by Poort S.R. and his colleagues in 1996 [1]. It represents the nucleotide replacement of guanine (G) with adenine (A) in the 3-untranslated region of the gene (20210), which leads to an increase in the prothrombin level in blood plasma by 1.5–2 relative to the normal range [1–4]. Prothrombin, or factor II, a vitamin K-dependent glycoprotein zymogen, is known to be a precursor of thrombin, which turns into thrombin under the influence of activated Factor X coagulation. Prevalence of prothrombin G20210A mutation depends on ethnicity and ranges from 0.7 to 6.7% [5–7].

Currently, there is good evidence regarding the association of GA genotype with the risk of thromboses [8–10] occurring due to an increase in both the level and activity of prothrombin in blood plasma [1, 11, 12].

It is still questionable whether there is association of prothrombin G20210A mutation with the risk of developing pregnancy complications. According to a series of meta-analyses and systematic reviews, association of the mutation with the risk of pregnancy complications is obvious and proven. For example, the heterozygous GA variant is associated with an increase in the risk of early pregnancy loss (EPL) by 2.5–2.7, PE by 2.5–7.1, FGR by 2.5–4.1 and preterm placental abruption by 4–8 [13–15]. However, other studies deny that association [16, 17]. It should be noted that in the works presented only the genotype was taken into account, and its phenotypic manifestation represented by an increase in prothrombin activity was not considered. Meanwhile, it is obvious that the previously described vascular microthromboses of the placental bed in women with genetic thrombophilia [18–21] are to be associated with a hypercoagulable shift due to the excessive activity of Factor II [22].

Lack of data on the level of prothrombin activity, presumably initiating the development of pregnancy complications in women with the GA genotype, was the reason for the present study.

### Objective

To study the relationship between the activity of prothrombin in blood plasma and the development of pregnancy complications in women with prothrombin G20210A mutation.

## Materials and methods

### Study population

A prospective clinical cohort study of 290 female patients was conducted within the clinical units of the FSBEI HE ASMU of the Ministry of Health of the Russian Federation from 2012 to 2018. Two groups were singled out: the study group of 140 patients with the GA genotype (average age 31.2 ± 4.7 years) and the control group of 150 women with the “wild” GG genotype (average age 32.3 ± 3.9 years).

A distinctive feature of our study was that we analyzed all pregnancy cases of the registered patients, not a single episode of gestation. So, in our opinion, it is of fundamental importance, since often a woman’s reproductive plans are not limited to the birth of one child, and it is important to understand the combination of which factors can lead to obstetric complications in carriers of the prothrombin G20210A genotype both in the first and subsequent pregnancies. Considering the above, during the observation period, the course and outcome of 814 pregnancies were analyzed: 363 in the group of the prothrombin G21210A mutation carriers and 451 in the control group. The association with the carriage of the prothrombin G20210A genotype was studied only in the case of planned pregnancies; artificial abortions were excluded.

Under complications of pregnancy we meant the following conditions: reproductive losses before 12 weeks of gestation, development of PE, FGR, and antenatal fetal death. Under non-developing pregnancy we understood anembryonic pregnancy - the absence of an embryo in a fetal egg or the death of an embryo. Preeclampsia and fetal growth retardation were diagnosed according to the criteria of international consensus [23, 24].

### Inclusion criteria

Criteria for the inclusion in the study group: prothrombin G20210A mutation, 18–45 years of age; singleton progressive pregnancy occurred without hormone stimulation; no abnormalities in the development of internal genital organs; no decompensated extragenital diseases; informed consent of the woman to be the subject of additional research methods. Criteria for the inclusion in the control group were the same as for the study group; however, the patients were not prothrombin G20210A mutation carriers.

### Exclusion criteria

Factor V Leiden mutation [F5L (1691)GA]; decreased functional activity of antithrombin and proteins C or S; genital malformations; multifetal pregnancy; pregnancy resulting from assisted reproductive technologies; decompensated extragenital diseases; autoimmune diseases, including antiphospholipid syndrome; chromosomal aberrations in spouses.

The clinical characteristics of the comparison groups according to traditional risk factors for the development of placental dysfunction are presented in Table 1.

**Table 1:** Clinical characteristics of patients admitted to the study

Variable		The study group, *GA genotype* *n* = 140		The control group *GG genotype* *n* = 150		Statistical value		
		absolute number	(%)	absolute number	(%)	OR	95%CI	p
Age	18–35 years old	101	72.1	112	74.7	0.9	0.521–1.480	0.6268
	> 35 years old	39	27.9	38	25.3	1.1	0.675–1.917	0.6268
Caucasian race		130	92.9	138	92.0	1.1	0.472–2.705	0.7831
BMI (kg/m2)	< 18.5	3	2.1	2	1.3	1.6	0.266–9.844	0.6
	18.5–25	98	70.0	112	74.7	0.8	0.472–1.32	0.3747
	≥25	25	17.9	30	20.0	0.6	0.482–1.567	0.8696
	≥30	8	5.7	4	2.7	2.1	0.651–7.516	0.2033
	≥35	6	4.3	2	1.3	3.3	0.657–16.698	0.1466
Hypertensive disease		34	24.3	13	8.7	3.4	1.699–6.723	0.0005

The groups were comparable by age (p > 0.05) and ethnicity: 93.2% of the study group and 91.9% of the control group were represented by Caucasians (*p* > 0.05). The body mass index of the patients also did not have significant differences. It should be noted that before the reproductive function was performed, the number of patients suffering from essential hypertension was comparable [OR = 1.8, 95% CI: 0.8926–3.8746; *p* = 0.0976]. By the end of the observation, the number of hypertension cases in the group of G20210A genotype was registered 3.4 times more often than in the control group. Upon that, in 13 women hypertension was the result of PE.

The study was approved by the local ethics committee of the FSBEI HE A**S**MU of the Ministry of Health of the Russian Federation (protocol No. 5 of June 25, 2012).

Preeclampsia and fetal growth retardation were diagnosed according to international consensus criteria [23, 24].

### Laboratory assays

All patients were diagnosed with prothrombin G20210A mutation and prothrombin activity, none of them receiving anticoagulants. Eight time points were selected to evaluate prothrombin activity, based on trophoblast invasion waves and major pregnancy periods: 7–8 weeks, 12–13 weeks, 18–19 weeks, 22–23 weeks, 27–28 weeks, 32–33 weeks, 36–37 weeks and 2–3 days after delivery [25, 26].

Prothrombin G20210A mutation was diagnosed by means of the polymerase chain reaction (PCR) method using reagents from Litekh SPA (Russia). Material for the study was human genomic DNA isolated from peripheral blood leukocytes. The analysis was based on the Real-Time PCR method using competing TagMan probes complementary to the polymorphic DNA sequence. In all patients, prothrombin activity was measured using Factor II deficient plasma (Siemens) on an automatic coagulometer (Siemens BCS XP) according to the previously described method [27].

### Statistics

Statistical data processing was performed using the MedCalc Version 17.9.7 statistical software package (license BU556-P12YT-BBS55-YAH5M-UBE51). Variation series were checked for normal distribution using the Shapiro-Wilk W-test. Laboratory values are presented as scatter plots with box plots (box-and-whisker plot). The box plot represents a median (Me) - middle of the sample, shown as a marker on the inside line of each box; interquartile range - interval between the 25th and 75th percentiles containing the central 50% of the sample’s observation, shown as a box; 95% confidence interval (95% CI) for the median shown as straight lines (whiskers) coming out of the box.

To compare the levels of prothrombin activity in two independent samples, the Mann-Whitney nonparametric statistical U-test was used. To determine the prognostic value of prothrombin activity index for the development of pregnancy complications in prothrombin G20210A mutation carriers, the ROC curve was used, with subsequent AUC calculation.

For qualitative features, the total and relative values were given in percentage; verification of statistical hypotheses on the coincidence of the observed and expected frequencies was performed using the χ2 criterion and Fisher’s exact test. For binary features, the relative risk (RR) and 95% confidence interval (95% CI) were calculated. The critical significance level of discrepancies (p) was defined as *p* < 0.05.

## Results

Analysis of the course and outcomes of planned pregnancies in the study groups showed that pregnancy complications were recorded in 57.9% (183 out of 316) cases with prothrombin G20210A mutation and in 25.1% (102 of 406) cases without it [RR 2.3; 95% CI 1.7–3.1; *p* < 0.0001], which is statistically significant (Table. 2).

**Table 2:** Reproductive history of patients admitted to the study

Clinical manifestation of prothrombin G20210A mutation	Study group *n* = 140		Control group *n* = 150		Statistics	
	total	%	total	%	p	OR. 95%CI
Total number of pregnancies	363	–	451	–	> 0.05	–
Artificial abortion	47	12.5	46	10.2	0.2	1.3 (0.9–1.9)
Planned pregnancy	316	87.1	406	90.0	0.2	0.97 (0.9–1.0)
Reproductive losses up to 12 weeks	92	9.1	47	11.6	< 0.0001	2.5 (1.8–3.5)
non-developing pregnancy	53	16.8	11	2.4	< 0.0001	6.2 (3.2–11.7)
spontaneous miscarriage	32	10.1	31	7.6	0.2	1.3 (0.8–2.1)
ectopic pregnancy	7	1.9	5	1.1	0.2	2.2 (0.7–7.6)
Favorable pregnancy	133	42	304	74.6	< 0.0001	1.8 (1.5–2.0)
Total number of deliveries	207	65.5	359	88.4	< 0.0001	1.3 (1.2–1.5)
Preterm delivery	30	9.5	6	1.5	< 0.0001	6.4 (2.7–15.2)
Fetal growth restriction	21	6.6	16	3.9	0.1	1.7 (0.9–3.2)
Preeclampsia	26	8.2	13	3.2	0.004	2.6 (1.3–4.9)
severe preeclampsia	15	4.7	2	0.5	0.003	9.6 (2.2–41.8)
Antenatal fetal death	6	1.9	1	0.2	0.06	7.7 (0.9–63.7)

The data analysis showed that the GA genotype is associated with the development of early reproductive losses in the form of a non-developing pregnancy and preeclampsia. It can be seen that all episodes (*n* = 30) of preterm delivery were accompanied by PE and/or FGR. All antenatally dead fetuses were diagnosed with growth retardation, and in 83.3% (5 out of 6), fetal death occurred against the background of preeclampsia.

**Fig. 1:**
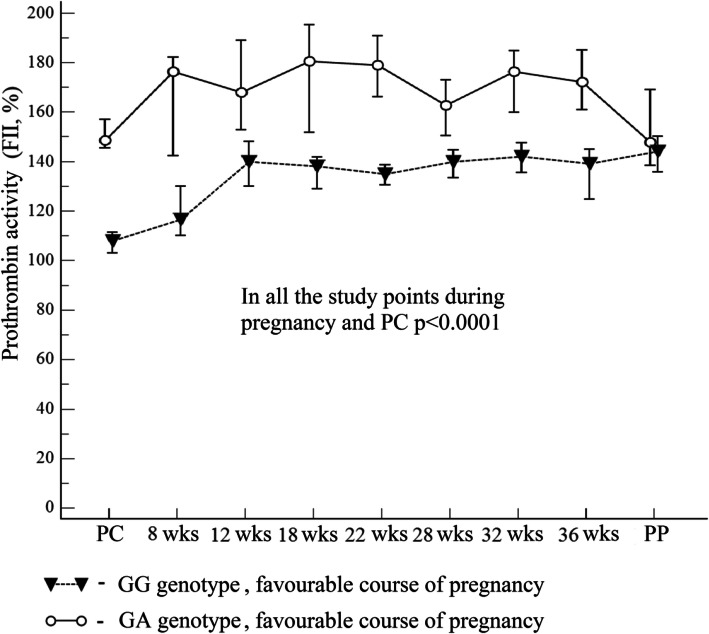
The level of prothrombin activity, depending on the genotype, at the preconception stage, at different gestational periods and postpartum. The results are given based on the previously published data obtained during normal pregnancy [28]

In the study, prothrombin activity in blood plasma was studied in 140 women with prothrombin G20210A mutation. The data obtained were compared to the results in pregnant women with the wild type (*n* = 40). It was found that the median of prothrombin activity at the points of study during normal pregnancy in the control group (GG genotype) ranged from 108% (preconceptional period) to 144% (pregnancy) (95% CI 130–150). At the same time, in pregnant women with the GA genotype, regardless of pregnancy course, it was significantly higher, ranging from 149 to 181% (95% CI 142–195) (Fig. 1).

**Fig. 2:**
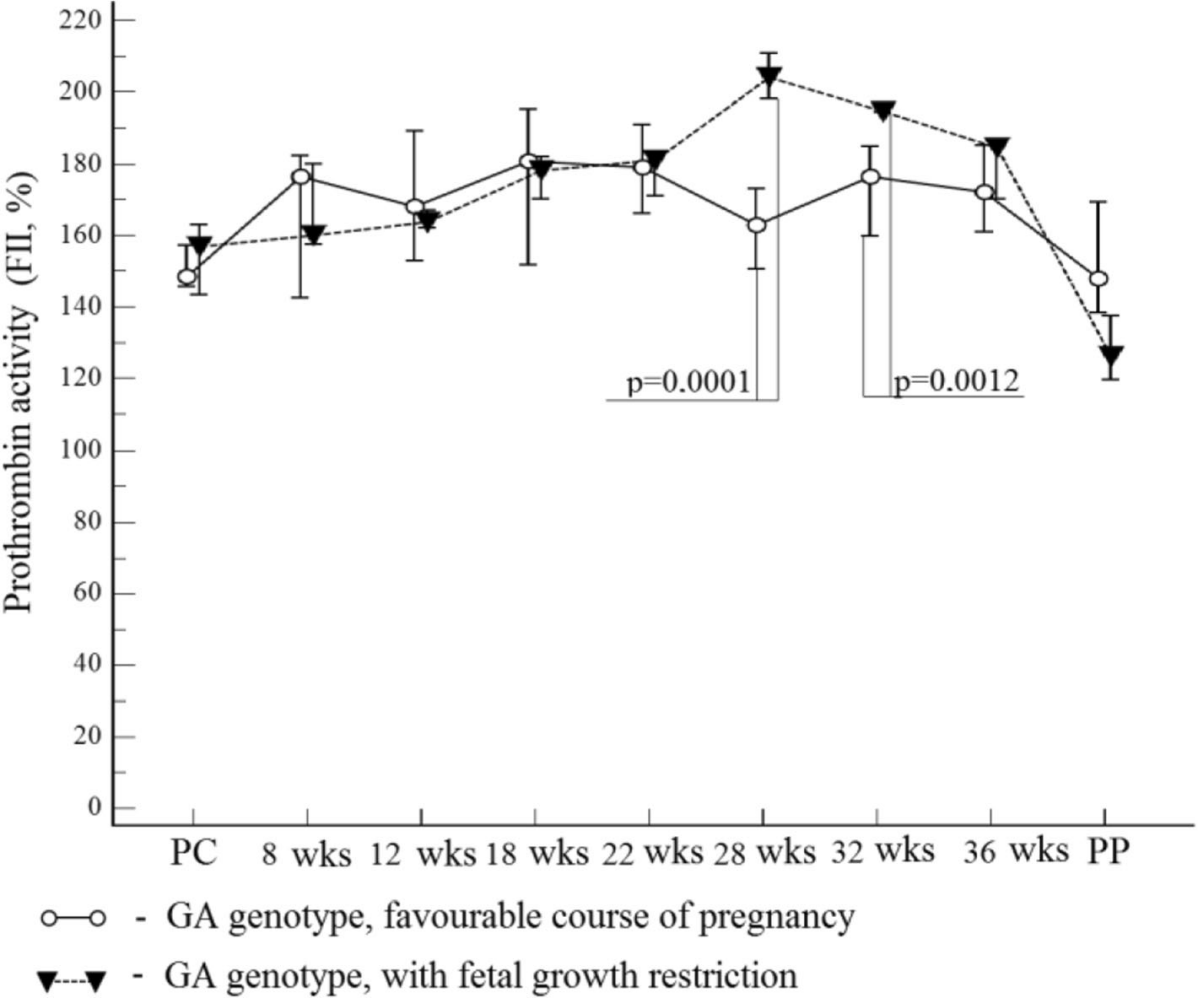
Median prothrombin activity (%) in GA genotype carriers with a favorable pregnancy and with FGR. The most significant results were obtained in the analysis of laboratory phenotype with early and/or severe preeclampsia in GA genotype carriers. Prothrombin activity in that group was significantly higher than in those with a favorable pregnancy starting from the preconception stage (Fig. 3)

**Fig. 3:**
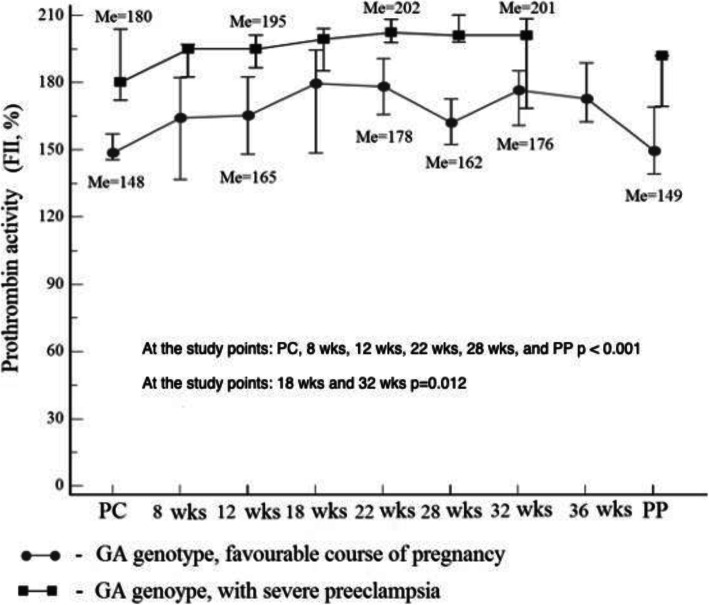
Median prothrombin activity (%) in GA genotype carriers with a favorable pregnancy and with early and/or severe preeclampsia

Here, as well as in Figs. 2 and 3: PC – preconception period; PP – postpartum on the 2nd – 3rd days. Median – marker; the values corresponding to 95% confidence interval are the lower and upper vertical bars (error bar).

In 103 (73.6% of 140) patients of the study group, prothrombin activity at the time points was from 148.5 to 180.6% to, which was associated with a favorable course and outcome of pregnancy. In 8 patients (5.7% of 140), pregnancy ended in fetal death at the gestational age of 8–9 weeks, while the median activity of prothrombin was 198.1% (95% CI 191.5–201.4), which is significantly higher than the same value at 8 weeks with developing pregnancy – 176.3% (95%CI 142,2-180,1) (*p* = 0.0069). With FGR before the gestational age of 22 weeks, prothrombin activity was comparable to that in women with a favorable pregnancy. At the gestational age of 28 weeks, a multidirectional change in prothrombin activity was recorded relative to 22 weeks: with favorable pregnancy, it decreased by 10.2% with the median of 162.0%, and with FGR it increased by 11.3%, with the median of 204.0%. The statistical difference between the medians of prothrombin activity remained at the gestational age of 32 weeks as well (176.4 and 194.7%, respectively) (Fig. 2).

It should be noted that all women (*n* = 15) had an assisted premature delivery within 28–32 weeks, given the severity of preeclampsia.

Considering the obtained results, in order to determine the prognostic significance of prothrombin activity for the development of early and/or severe preeclampsia from the preconception period or early pregnancy, ROC analysis was performed [29].

Table 3 presents the summary data of ROC analysis for the level of prothrombin activity at the points of study, as a prognostic marker for the development of early and/or severe preeclampsia.

**Table 3:** The results of ROC analysis to predict early and/or severe preeclampsia at different stages of pregnancy using the level of prothrombin activity as a marker for patients with prothrombin G20210A mutation

Statistical values	PC (*n* = 15)	7–8 weeks *n* = 15	12–13 weeks *n* = 15	18–19 weeks *n* = 15	22–23 weeks *n* = 14	27–28 weeks *n* = 11	32–33 weeks *n* = 10
Prothrombin activity cutoff (%)	> 171.0	> 181.3	> 180.0	> 180.6	> 191.5	> 196.0	> 192.1
Sensitivity	80	93.3	95	100	100	100	72.7
Specificity	87.8	70	71	55.6	69.2	93.3	75
Area under ROC curve (AUC)	0.863	0.840	0.792	0.756	0.788	0.958	0.741
95% CI for AUC	0.779–0.925	0.677–0.942	0.609–0.916	0.575–0.888	0.654–0.889	0.844–0.996	0.605–0.849
(p)	< 0.0001	< 0.0001	0.0022	0.0031	< 0.0001	< 0.0001	0.002

**Fig. 4:**
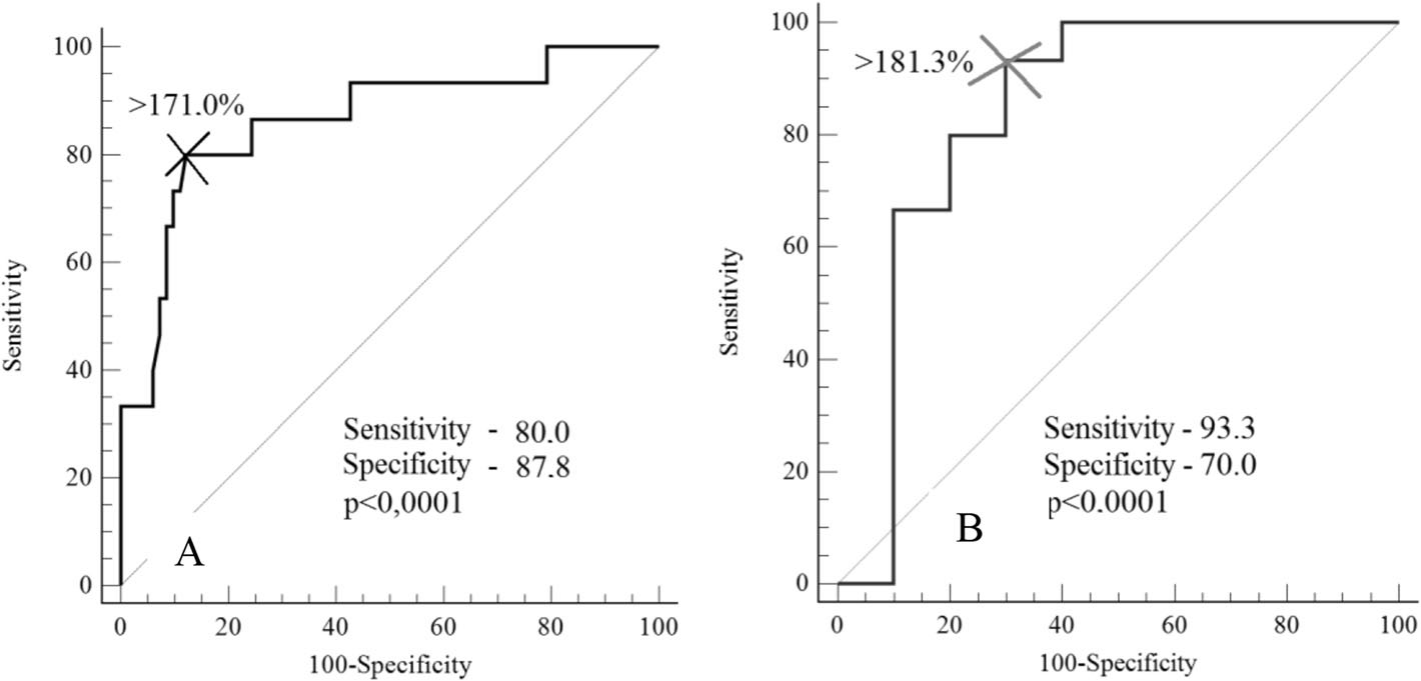
ROC curves of the model to predict preeclampsia by prothrombin activity level (%) for patients with prothrombin G20210A mutation. A – at the preconception stage; B – at the gestational age of 7–8 weeks

Thus, in predicting preeclampsia based on the prothrombin activity level, ROC analysis made it possible to determine the optimal cut-off threshold with the best predictive ability, which was > 171.0% at the preconception stage and > 180.0% at all stages of pregnancy. The area under the ROC curve (AUC) in all points of the study showed good prognostic power and clinical significance of the method (Table 3). At the same time, the risk of developing early and/or severe preeclampsia at the preconception stage is predicted in 86% of cases and at the gestational age of 7–8 weeks in 84% of cases. The graphic representation of ROC curves with the maximum test performance is shown in Fig. 4.

## Discussion

The results of the present study illustrate an association between prothrombin G20210A mutation and the risk of early reproductive loss (OR 2.5), preeclampsia (OR 2.6) including early and/or severe preeclampsia (OR 9.6), and preterm birth rates (OR 6.4) – see Table 1. The demonstrated risks are comparable with the results of some previously submitted studies in this field [10, 13, 14].

It should be noted that the GA genotype did not affect the frequency of spontaneous miscarriages up to 12 weeks (OR 1.3; *p* = 0.2), while increasing the risk of non-developing pregnancy with statistical significance (OR 6.2). All episodes of early reproductive losses in the study were due to fetal death within the period of 8–9 weeks, which is consistent with the data by other authors [15, 30, 31].

Aetiopathogenesis of a non-developing pregnancy is known to be multifaceted, and one of its components is microthrombosis of placental bed vessels [21, 32]. In our study, such thrombosis can be caused by an increased level of prothrombin activity, determined at the gestational age of 7–8 weeks. However, the number of cases (*n* = 8) does not seem sufficient to extrapolate the findings, so further research is required.

We agree with a number of authors that prothrombin G20210A mutation is by no means always accompanied by thrombosis and/or gestational complications [17, 33]. We nevertheless believe that the development of a clinically significant event (thrombosis and/or placenta-mediated complications) in the considered cases is preconditioned by an over-threshold level of prothrombin activity. Such a pattern was previously described for thrombosis [12, 34].

Demonstration of the association between phenotypic manifestation of the GA genotype represented by a significant increase in prothrombin activity and pregnancy complications is the key point of the study. We have defined different dynamics of prothrombin activity from the preconception period and throughout pregnancy, depending on the development of FGR or PE, which makes it possible to consider this activity as a prognostic marker of PE development, starting from the preconception period.

## Conclusion

Studying the level and dynamics of prothrombin activity during the development of gestational complications makes it possible to change the view on the stratification of the prognosis of pregnancy complications in women with prothrombin G20210A mutation. Prothrombin activity can be considered a prognostic marker for the development of preeclampsia with the greatest accuracy at the preconception stage (AUC – 0.86; *p* < 0.0001) and at the gestational age of 7–8 weeks (AUC – 0.84; p < 0.0001). The revealed patterns can be promising for personalized medicine in terms of considering the feasibility of heparin prophylaxis in the settings of prothrombin G20210A mutation.

## Data Availability

The research protocol, statistical analysis plan, analysis principles and data on individual participants that underlie the results presented in this article after de-identification (text, tables) will be available at the request of researchers who will provide a methodologically reasoned proposal for a meta-analysis of individual participants’ data 9 months later and up to 3 years after the publication of the article. Proposals should be sent to the e-mail nikolmg@yandex.ru. In order to gain access, data requesters will need to sign a data access agreement.

## Abbreviations

AUC: Area under the curve
APC-R: activated protein C resistance
95%CI: 95% confidence interval
EPL: Early pregnancy loss
FGR: Fetal growth retardation
F2 (20210) GA: Prothrombin G20210A mutation (GenBank 176,930.0009)
F5L (1691) GA: Factor V Leiden mutation (GenBank 612,309.0001)
Ме: Median
NR: Normalized ratio
р: The significance level of differences
OR: Оdds Ratio
PE: Preeclampsia
ROC: Receiver operating characteristic
RR: Relative Risk
